# The Effect of Interactions of Single Nucleotide Polymorphisms of APOA1/APOC3 with Food Group Intakes on the Risk of Metabolic Syndrome

**Published:** 2017

**Authors:** Firoozeh Hosseini-Esfahani, Parvin Mirmiran, Maryam S. Daneshpour, Azadeh Mottaghi, Fereidoun Azizi

**Affiliations:** 1. Nutrition and Endocrine Research Center, Research Institute for Endocrine Sciences, Shahid Beheshti University of Medical Sciences, Tehran, Iran; 2. Faculty of Nutrition Sciences and Food Technology, National Nutrition and Food Technology Research Institute, Shahid Beheshti University of Medical Sciences, Tehran, Iran; 3. Cellular Molecular and Endocrine Research Center, Research Institute for Endocrine Sciences, Shahid Beheshti University of Medical Sciences, Tehran, Iran; 4. Endocrine Research Center, Research Institute for Endocrine Sciences, Shahid Beheshti University of Medical Sciences, Tehran, Iran

**Keywords:** Food, Genetic polymorphisms, Metabolic Syndrome

## Abstract

**Background:**

The aim of this study was to examine the interaction of dietary food groups and genetic variants of APOA1/APOC3, relative to Metabolic Syndrome (MetS) risk in adults.

**Methods:**

In this matched nested case-control study, 414 MetS subjects and 414 controls were selected from among participants of Tehran Lipid and Glucose Study. Dietary intake was assessed with the use of a valid and reliable semi-quantitative food frequency questionnaire. Single Nucleotide Polymorphisms (SNPs), APOA1 (rs670, −75G>A and rs5069, +83C>T/APOC3 rs5128 C3238>G) were genotyped by the conventional polymerase chain reaction and restriction fragment length polymorphism.

**Results:**

The mean (SD) of age was 40.7 (13) and 41.2 (13) years in male cases and controls versus 44.0 (11) and 44.0 (12) years in female case and controls. A significant interaction between intake quartiles of the sugar group and APOA1 combined group (GA+AA/CT+TT) SNPs was found; The ORs for these genotype carriers were (1, 0.44, 0.36, 0.23; P trend<0.001) in quartiles of intake, relative to other combined genotypes (P interaction=0.02). MetS risk appeared to be increased significantly in higher quartiles of sweet beverages and fish intakes in the GA+AA/CT+TT/CC genotypes of APOA1/APOC3 SNPs, compared to other genotypes (P interaction=0.01). The combined effect of genotypes of APOC3/APOA1 showed further decrease in MetS risk in higher quartiles of sugar group intakes (OR: 1, 0.24, 0.26, 0.14, P trend=0.001) relative to other combinations (P interaction=0.008).

**Conclusion:**

Results obtained demonstrate that some dietary food groups (sugar, fish, and sweet beverages) modulate the effect of APOA1/APOC3 SNPs in relation to MetS risk.

## Introduction

The worldwide increase in the incidence of the Metabolic Syndrome (MetS) and diabetes mellitus over the last few decades is a salient example of how the interaction between lifestyle and genotype can dramatically impact health. Some of good reasons for this interaction are the familial nature of MetS ^1,2^, the obvious difference in the prevalence of MetS within various racial groups ^3^ and the different rates of MetS prevalence among monozygotic twins ^4^. Interactions between genes and diet play important roles in the development of MetS, although contribution of each per se can be different ^5–7^. For example, in a study conducted on 303 elderly pairs of twins, it was observed that glucose intolerance, obesity and low concentrations of High Density Lipoprotein Cholesterol (HDL-C) are significantly higher among monozygotic twins than among dizygotic twins, indicating the outstanding role of genetics in this area. On the other hand, in the case of insulin, triglyceride levels and blood pressure, the role of environmental factors including diet were more important than genetics ^4^.

Decreased HDL-C concentration is a hallmark of the dyslipidemic profile associated with the development of MetS ^8^. Apolipoprotein A1 (APOA1) is one of the major structural and functional protein constituents of HDL-C and an *in vivo* activator of Lecithin-Cholesterol- Acyltransferase (LCAT). An inverse relationship has been reported between apoA1 and HDL-C and CVD in general populations ^9,10^. The gene encoding APOA1 is highly polymorphic and several Single Nucleotide Polymorphisms (SNPs) have been identified in the apoA1 gene located on the long arm of chromosome 11, in association with plasma lipid and lipoprotein concentrations. Among these, a common G/A substitution located at –75 *bp* upstream from the apoA1 transcription start site (rs670) and a C/T substitution at position +83 *bp* (rs5069) from the first intron were reported to differentially affect apoA1 expression ^11^. Some studies have shown that allele A is strongly associated with higher HDL-C and APOA1 ^12,13^. Moreover, MetS risk was exacerbated among individuals who had high intakes of fat (>35% energy) and monoun-saturated fat (>14% energy) and GG homozygote for the APOA1 rs670 ^14^. The relevance of APOC3 to human triglyceride (TG) rich lipoprotein metabolism comes primarily from genetic studies, which have consistently shown associations between the APOC3 locus and TG concentration. APOC3 inhibits lipolysis of very low-density lipoproteins via non-competitive inhibition of lipoprotein lipase and hepatic triglyceride lipase activity, and by interfering with lipoprotein binding to glycosaminoglycans on cell membranes ^15^. Of several variants within APOC3, a transversion from C to G in the 3′ untranslated region (3′UTR) of exon 4 results in an rs5128 polymorphism (also known as SstI, sacI, 3238C>G or 3175C>G) ^16^. One study showed that higher levels of Low Density Lipoprotein Cholesterol (LDL-C) and total cholesterol were associated with a higher saturated fatty acid intake for the G allele of APOA1 G-75A (rs670) and higher LDL-C was associated with a higher saturated fat and total fat intake for allele C of APOC3 3238C>G (rs5128) ^17^. Due to the fact that until now, no study has investigated interactions between food groups and APOA1/APOC3 SNPs, the main goal of present study was to examine the interaction of dietary food groups and some genetic variants of APOA1/APOC3, relative to MetS risk in adults.

## Materials and Methods

### Study population

This nested case-control study was a part of the Tehran Lipid and Glucose Study (TLGS), an ongoing population based cohort study being conducted to determine the risk factors of non-communicable diseases on a sample of residents of district 13 of Tehran, the capital of Iran. The baseline examination survey was conducted from 1999 to 2001 in 15005 subjects, aged ≥3 years, and follow-up examinations have been conducted every 3 years (2002–2005; 2006–2008; 2008–2011) to update health related measurements of baseline characteristics and to identify any newly developed diseases ^18^.

Of 11001 and 9807 individuals, aged≥18 years, who participated in baseline and second follow-up surveys respectively, 5280 were excluded because of having MetS at either baseline or second follow-up survey. In the current study, 503 cases were randomly selected from among participants who developed MetS at the third (n=918) and fourth (n=827) follow-up surveys. After excluding individuals with a history of cardiovascular events, weight loss or gain >5 *kg* in the last 6 months, pregnant or lactating women, or those taking any CVD/anticoagulant/steroid or hormonal medication (n=20), 483 cases were included in the study. Controls were defined as participants with ≤1 MetS components at the time that the corresponding case developed MetS. Each case was individually pair matched with a control, randomly by age (±5 years) and sex. After excluding cases/controls lacking DNA purification in the range of 1.7<A260/A280<2(n=108), and those whose reported energy intake divided by the predicted energy intake did not qualify for the ±3 SD range (n=30), finally, 828 (414 pairs) MetS and matched controls remained for the analysis. The study protocol was approved by the ethics committee of the Research Institute for Endocrine Sciences, Shahid Beheshti University of Medical Sciences, Tehran, Iran. Written informed consent was obtained from each participant.

### Dietary intakes

Dietary intake was assessed with the use of a valid and reliable, 168-item, semi-quantitative food frequency questionnaire (FFQ), which included a list of foods with standard serving sizes. Based on macronutrient composition and using available literature, 25 food groups were categorized ^19–21^ including different kinds of green, red-yellow, starchy and other vegetables (squash, eggplant, onions, garlic, cabbages, peppers and chilli), fruits (all kinds of fruits and dried fruits), nuts and seeds, legumes, eggs (all kinds of preparations), liquid oils, solid oils (butter and hydrogenated fats), low fat dairy, high fat dairy, whole grains (whole wheat breads, flours and biscuits, barley, wheat, corn and sprouts), refined grains (rice, macaroni, white bread and flours), fast foods (processed meats, fried potato and pizza), soft drinks (all kinds of sugar added beverages), salty snacks (salty biscuits, pickles and chips), red meats, organ meats, fish(all kinds of preparations, tuna fish), poultry (all kinds of preparations), sweets (cakes, donut, biscuits, desserts and chocolates), sweetened fruit juices, sugar (sugar, jam, honey, candy), tea and coffee.

### Anthropometric measurements and blood pressure

While the subjects were minimally clothed and not wearing shoes, weight was measured using digital scales (Seca 707; Hamburg, Germany) and recorded to the nearest 100 *g*; Height was measured with a tape measure (model 208 Portable Body Meter Measuring Device; Seca) while the subjects were standing with shoulders in normal alignment and not wearing shoes. Body Mass Index (BMI) was calculated. Waist Circumference (WC) was measured at the umbilical level with the use of an upstretched tape measure without any pressure to body surface. Measurements were recorded to the nearest 0.1 *cm*. Blood Pressure (BP) was measured twice, at least 30-second interval, after the participants sat for 15 *min* and the mean BP of the 2 measurements recorded was reported as the subject’ s BP.

### Physical activity

Physical activity level was assessed using the Persian translated Modifiable Activity Questionnaire (MAQ) in Tehranian adults, for which high reliability and relatively moderate validity have been reported. The frequency and time spent on light, moderate, hard and very hard intensity activities according to the list of common activities of daily life over the past year were obtained. Physical activity levels were expressed as metabolic equivalent hours per week (METs/h/wk)^22–24^.

### Laboratory assays

Between 7:00 and 9:00 AM, after 12 to 14 *hr* of overnight fasting, blood samples were drawn into vacutainer tubes in a sitting position from all study participants ^18^. Samples were centrifuged within 30 to 45 *min* of collection according to standard protocols. All biochemical analyses were performed at the TLGS research laboratory on the same day of blood collection, and analyses were conducted using a Selectra 2 autoanalyzer (Vital Scientific, Spankeren, the Netherlands). Fasting Blood Glucose (FBG) was measured by the enzymatic colorimetric method using glucose oxidase. Triglycerides (TG) were measured using TG kits (Pars Azmoon, Tehran, Iran) by enzymatic colorimetric tests and with glycerol phosphate oxidase. HDL-C was measured after precipitation of apolipoprotein B containing lipoproteins with phosphotungstic acid. Monitoring of assay performance was performed once every 20 tests, using lipid control serum, Percinorm (reference range), and Percipath (pathologic range) wherever applicable (Boehringer Mannheim, Germany; catalog no. 1446070 for Percinorm and 171778 for Percipath). Lipid standard (Cfas, Boehringer Mannheim; catalog no. 759350) was used to calibrate the Selectra 2 auto analyzer for each day of laboratory analyses, and all samples were analyzed when internal quality control met the acceptable criteria. Inter- and intra-assay coefficients of variation were both 2.2% for serum glucose and 1.6% and 0.6% for TG, respectively.

### Genetic analysis

For genotyping the APOA1/APOC3 polymorphisms, buffy coats were separated from the non-coagulated whole blood samples and stored at −70 °*C* until processing, when genomic DNA was extracted by the Proteinase K, salting out method. The Polymerase Chain Reaction followed by Restriction Fragment Length Polymorphism technique (PCR-RFLP) was employed to investigate polymorphisms in the gene fragments ^25,26^.

PCR was done using the following primer; APOA1 (rs670, −75G>A and rs5069,+83C>T) (F: 5′-AGG GAC AGA GCT GAT CCTTGA ACT CTT AAG-3′; R: 5′-TTA GGG GCA CCT AGCCCT CAG GAA GAG AGC A-3′)/APOC3 (rs5128, C3238>G) (F:5′-GGT GAC CGA TGG CTT CAG TT-3′; R:5′-CAGAAG GTG GAT AGA GCG CT-3′); hybridization was carried out in a DNA thermal cycler (Corbett co. Australia). Amplified DNA was digested with MspI (HpaII) and SacI restriction enzymes (Fermentas) at 37 °*C* for overnight ^27^; fragments were separated by electrophoresis on a 2% agarose gel. DNA fragments were visualized by gel documentation (Optigo Co. Optigo, Ijsselstein, The Netherland); 5% of samples were randomly replicated and genotyped with ≥99% concordance. Five percent of the PCR samples were directly sequenced to confirm the PCR-RFLP results.

### Definitions

MetS was defined as participants with three or more of the following conditions by the modified National Cholesterol Education Program/Adult Treatment panel III (ATP III) definition ^28,29^: 1) TG ≥150 *mg/dl* or drug treatment, 2) Low density lipoprotein cholesterol (LDL-C) <40 *mg/dl* in men and <50 *mg/dl* in women or drug treatment; 3) Elevated BP ≥130/85 *mmHg* or drug treatment for a previous diagnosis of hypertension, and 4) FBG ≥110 *mg/dl* or drug treatment of hyperglycemia. WC was coded according to the newly-introduced cut-off points for Iranian adults, *i.e*. ≥95 *cm* for both genders.

### Statistical analysis

Statistical analyses were performed using the Statistical Package for Social Sciences (Version 16.0; SPSS, Chicago, IL) and STATA (Statistics/Data analysis 12.0). A 2-sided p-value <0.05 was considered significant. Pearson’s chi-square statistic was used to test the Hardy-Weinberg Equilibrium for the SNP and to test the differences in percentages, using Power-Marker software. TG concentration was log-transformed for the statistical analysis. Multiplicative interactions between quartiles of food group intakes and polymorphisms (dominant model) in relation to MetS were examined using conditional logistic regression analysis, after adjustment for baseline BMI and using the likelihood ratio test; a comparison of the likelihood scores of the 2 models with and without the interaction terms. Conditional logistic regression was used to examine the joint role of quartiles of dietary pattern scores and genotypes of rs5128 (CC/CG+GG), rs670 (GG/GA+AA) and rs5069 (CC/CT+TT) in predicting MetS risk, using the lowest quartile of food group in-takes and homozygote group with the major allele as the reference category. For combined analyses, the risk genotypes, which comprised the rs670 (GA+AA) and rs5069 (CT+TT) were included in the analysis. When APOC3 rs5128 was included in the analysis, the risk genotype combination comprised rs670 (GA+AA), rs5069 (CT+TT) and rs5128 (CC). Conditional logistic regression was used to examine the joint role of quartiles of food group intakes and genotype combinations in predicting MetS risk, which were adjusted for baseline BMI. To determine the p-value for trend across the quartiles of food group intakes, logistic regression was used with the median of each quartile of food group intakes used as a continuous variable.

## Results

The mean (SD) age of study participants was 42.1 (12) and 42.4(13) years in cases and controls at baseline, respectively. Cases had higher mean BMI in comparison with controls, 26.1(4) *vs*. 22.9(4) *Kg/m*^2^ (p< 0.05) at baseline. In MetS subjects, the mean (SD) of MetS risk factors were different with controls at baseline; the low HDL-C concentration (78.5%) and abdominal obesity (74.6%) had the highest prevalence among MetS risk factors. There were no statistical differences in the mean energy and macronutrient intakes, depending on MetS status (Table 1).

**Table 1.** Characteristics of the study population in subjects with metabolic syndrome (MetS) cases and controls: Tehran Lipid and Glucose Study

	**With MetS (n=414)**	**without MetS (n=414)**
**Baseline Age (y)**	42.1(12) ^†^	42.4(13)
**Men (n=470)**	40.7(13)	41.2(13)
**Women (n=358)**	44.0(11)	44.0(12)
**Current smokers (*%*)**	12.3	15.0
**Low physical activity (*%*)**	36.8	33.5
**Education level (≥14 years) (*%*)**	13.2	16.3
**Baseline BMI (*Kg/m*^2^)**	26.1(4)	22.9(4) ^*^
**Obesity (*%*) ^1^**	38.6	3.6 ^*^
**Baseline WC (*cm*)**	86.2(11)	78.0(10) ^*^
**Abdominal obesity (*%*)^2^**	74.6	5.1 ^*^
**Baseline systolic BP (*mmHg*)**	114(12)	109(11) ^*^
**Baseline diastolic BP (*mmHg*)**	75.0(8)	71.5(8) ^*^
**Elevated BP (*%*)^3^**	43.8	2.2 ^*^
**Baseline HDL-C (*mg/dl*)**	39.3(10)	47.4(10) ^*^
**Low HDL-C (*%*)^4^**	78.5	1.4 ^*^
**Baseline TG (*mg/dl*)**	148(74)	101(44) ^*^
**High TG (*%*)^5^**	72.0	0.5 ^*^
**Baseline FBG (*mg/dl*)**	90.0(13)	86.6(10) ^*^
**High FBG (*%*)^6^**	9.4	1.0 ^*^
**Energy intake (*kcal/day*)**	23341070	2341(979)
**Carbohydrate (*%* of energy)**	58.9(7)	58.8(6)
**total fat (*%* of energy)**	29.9(6)	30.1(6)
**saturated fat (*%* of energy)**	9.8 (3)	10.6(10)
**monounsaturated fat (*%* of energy)**	10.2(3)	10.1(2)
**Fiber intake (*g*/1000 *kcal/d*)**	17.9(7)	18.1(7)

* P<0.05;

† values are mean (SD) unless otherwise listed. BMI: body mass index, WC: waist circumference, BP: blood pressure, HDL-C: high density lipoprotein cholesterol, TG: triglycerides, FBS: fasting blood glucose.

1 BMI≥30 *kg/m*^2^,

2 WC≥95 *cm* for genders,

3 Elevated BP ≥130/85 *mmHg*,

4 HDLC <40 *mg/dl* in men and <50 *mg/dl* in women;

5 TG≥150 *mg/dl*,

6 High FBG ≥110 *mg/dl*.

No statistically significant differences were observed in genotype frequencies of APOA1 rs670/rs5069 and APOC3 rs5128 between MetS subjects and controls. Genotype frequency did not deviate from Hardy-Weinberg equilibrium expectations, except for rs670 (Table 2).

**Table 2.** Genotype and allele frequency of APOA1 and APOC3 polymorphisms in subjects with metabolic syndrome (MetS) cases and controls: Tehran Lipid and Glucose Study

	**With MetS (n=414)**	**without MetS (n=414)**	**p-value**
**Allele frequency rs670**			0.12
G	589(71)^*^	604(73)	
A	239(29)	227(27)	
**Genotype frequency rs670**			0.62
GG	186(45)	197(47)	
GA	217(52)	210(51)	
AA	11(3)	7(2)	
**Allele frequency rs5069**			0.12
C	648(78)	654(79)	
T	180(22)	174(21)	
**Genotype frequency rs5069**			0.65
CC	258(62)	259(62)	
CT	132(32)	136(33)	
TT	24(6)	19(5)	
**Allele frequency rs5128**			0.20
C	645(81)	655(81)	
G	155(19)	157(19)	
**Genotype frequency rs5128**			0.76
CC	261(65)	261(44)	
CG	123(31)	133(33)	
GG	16(4)	12(3)	

* n(%)

Adjusted odds ratio for MetS according to quartile classification of food groups by the dominant model of APOA1/APOC3 genotypes are presented in table 3. The risk of MetS was not homogenous in APOA1 rs670 genotypes, across quartiles of sugar and nut group intakes; A allele carriers of rs670 had lower odds of MetS in higher quartiles of sugar group intake (OR: 3.0, 1.60, 1.52, 1.30, P trend<0.01) (P interaction= 0.02), compared to the GG genotype (OR: 1, 1.70, 1.31, 2.20, P trend=0.46). The GG genotype group of rs670 and high nut group intake had the highest MetS risk, compared with minor allele carriers and the low nut group intake (P interaction=0.0006). MetS risk appeared to be significantly increased in higher quartiles of fast food (P trend=0.04) and salty snack (P trend=0.006) group intakes in A (allele carriers of APOA1 rs670). Examination of other food groups did not identify a gene-food group interaction with APOA1 rs670 genotypes (data not shown).

**Table 3.** Adjusted ORs (95% CI)^1^ for metabolic syndrome according to quartile classification of food group intakes by dominant model of APOA1/APOC3 genotypes

	**Q1**	**Q2**	**Q3**	**Q4**	**P for trend**	**P for interaction**
**APOA1 genotypes (rs670)**						
**Fast foods**						0.08
**GG (rs670) (n=383)**	1 (ref.)	0.7 (0.35–1.56)	1.20 (0.57–2.44)	0.6 (0.27–1.22)	0.32	
**GA+AA (rs670) (n=445)**	0.7 (0.31–1.32)	1.10 (0.55–2.21)	1.20 (0.57–2.41)	1.30 (0.60–2.85)	0.04	
**Salty snacks**						0.06
**GG (rs670) (n=383)**	1	0.41 (0.19–0.87)	1.40 (0.64–2.85)	1.04 (0.50–2.16)	0.51	
**GA+AA (rs670) (n=445)**	0.69 (0.33–1.43)	1.07 (0.52–2.20)	1.23 (0.60–2.54)	1.34 (0.66–2.71)	0.006	
**Sugar**						0.02
**GG (rs670) (n=383)**	1	1.70 (0.87–3.44)	1.31 (0.64–2.77)	2.20 (1.02–4.48)	0.46	
**GA+AA (rs670) (n=445)**	3.0 (1.44–6.22)	1.60 (0.87–3.53)	1.52 (0.76–3.09)	1.30 (0.66–2.67)	<0.01	
**Nuts**						0.0006
**GG (rs670) (n=383)**	1	0.87 (0.38–1.64)	1.31 (0.65–2.63)	2.40 (1.13–4.91)	0.01	
**GA+AA (rs670) (n=445)**	1.80 (0.86–3.60)	1.82 (0.93–3.66)	0.80 (0.30–1.61)	1.92 (0.96–3.78)	0.31	
**APOA1 genotypes (rs5069)**						
**Fish**						0.04
**CC (rs5069) (n=517)**	1	1.34 (0.70–2.57)	1.07 (0.58–1.96)	0.63 (0.34–1.18)	0.75	
**CT+TT (rs5069) (n=311)**	0.48 (0.23–0.98)	1.14 (0.58–2.28)	0.96 (0.46–1.98)	1.30 (0.64–2.77)	0.20	
**Tea-coffee**						0.007
**CC (rs5069) (n=517)**	1	0.78 (0.41–1.47)	0.32 (0.16–0.61)	0.67 (0.36–1.27)	0.09	
**CT+TT (rs5069) (n=311)**	0.69 (0.34–1.43)	0.44 (0.21–0.92)	0.92 (0.42–1.96)	0.48 (0.24–0.98)	0.70	
**Sugar**						0.04
**CC (rs5069) (n=517)**	1	1.39 (0.76–2.55)	1.04 (0.54–2.01)	1.53 (0.84–2.81)	0.66	
**CT+TT (rs5069) (n=311)**	1.97 (0.94–4.13)	1.21 (0.59–2.49)	1.11 (0.55–2.23)	0.73 (0.36–1.47)	0.003	
**APOC3 genotype (rs5128)**						
**Salty Snacks**						0.15
**CC (rs5128) (n=522)**	1	0.78 (0.41–1.49)	1.17 (0.62–2.18)	1.73 (0.92–3.24)	0.02	
**CG+GG (rs5128) (n=284)**	0.75 (0.36–1.55)	0.66 (0.32–1.39)	1.73 (0.82–3.61)	0.76 (0.34–1.69)	0.26	

OR: Odds Ratio, Q: Quartile of food group intakes. APOA1: rs670 (−75G>A), rs5069 (+83C>T), APOC3: rs5128 (3238C>G)

1 ORs (95% CI) were calculated by using conditional logistic regression model, adjusted for baseline BMI and energy intake. Participants were joint classified (8 groups) according to quartiles (Q) of food group intakes and dominant model of rs5128, rs670 and rs5069 genotypes. The lowest quartile of food group intakes and homozygote genotype of major allele were used as the reference group.

There were interactions between APOA1 rs5069 and three food group intakes in relation to MetS risk: for the fish group intake, participants with T allele carriers had higher MetS risk when they had higher intakes of this group (P interaction=0.04). Carriers of CC genotype of rs5069 had lower MetS risk with high tea-coffee intakes, compared to those with lower intakes. There was a decreasing trend of ORs of MetS risk (OR: 1.97, 1.21, 1.11, 0.73, P trend<0.01) in T allele carriers of rs5069 and higher sugar group intakes, compared to the CC genotype (OR: 1, 1.39, 1.04, 1.53, P trend=0.66), (P interaction=0.04). MetS risk appeared to be significantly increased in higher quartiles of salty snack group intake in CC homozygote of APOC3 rs5128 (P trend=0.02). Examination of other food groups did not identify a gene-food group interaction with APOC3 genotypes (data not shown).

Adjusted ORs (95% CI) for MetS according to quartile classification of food group intakes by the combined model of APOA1/APOC3 genotypes were shown in table 4. The combined group of APOA1 SNPs (rs670/rs5069) interacted with sugar group intake in determining MetS (P interaction=0.02). The A and T allele carriers (GA+AA/CT+TT) in the highest quartile of sugar group intake had the lowest risk of MetS (OR: 0.23, CI: 0.08–0.64), compared to other combinations and lower sugar intake. A significant interaction between quartiles of the fish group intake and APOA1 combined group was found; the ORs for the A and T allele carriers were (1, 2.80, 2.41, 4.0; P trend=0.20) in quartiles of fish group intake, relative to other combined genotypes (2.60, 3.91, 2.90, 1.82; P trend=0.88) (P interaction=0.03). The combination of APOA1 genotypes did not alter the decreasing trend of MetS risk in quartiles of other food group intakes (data not shown). The combined effect of genotypes of APOC3/APOA1 showed further decrease in MetS risk in higher quartiles of sugar group intakes (OR: 1, 0.24, 0.26, 0.14, P trend=0.001) relative to other combinations (P interaction=0.008). The genotype carriers (GA+AA/CT+TT/CC) in the highest quartile of fish group intake had greater risk of MetS (OR: 6.27, CI: 1.86–21.2), compared to other genotypes. The SNPs×sweet beverage group intake was seen in relation to MetS risk. Carriers of GA+AA/CT+TT/CC genotypes and high intake of sweet beverage group showed further increase in MetS risk (OR: 1, 4.69, 7.62, 9.58, P trend<0.001) relative to other combinations (OR: 2.98, 3.93, 4.07, 4.41, P trend<0.009) (P interaction=0.03). Other food groups did not modulate the association of combined genotype carriers of APOA1 and APOA1/APOC3 in relation to MetS risk.

**Table 4.** Adjusted OR (95% CI)^1^ for metabolic syndrome and combined APOA1/APOC3 genotypes according to quartile classification of food group intakes

	**Q1**	**Q2**	**Q3**	**Q4**	**P for trend**	**P for interaction**
**APOA1 (rs670/rs5069)**						
**Sugar**						0.02
**GA+AA/CT+TT (n=229)**	1	0.44 (0.17–1.16)	0.36 (0.13–0.99)	0.23 (0.08–0.64)	0.001	
**Others (n=599)**	0.40 (0.17–0.92)	0.53 (0.23–1.24)	0.44 (0.19–1.03)	0.58 (0.25–1.33)	0.64	
**Fish**						0.03
**GA+AA/CT+TT (n=229)**	1	2.80 (1.10–6.94)	2.41 (0.87–6.77)	4.0 (1.45–10.88)	0.20	
**Others (n=599)**	2.60 (1.15–5.77)	3.91 (1.64–8.94)	2.90 (1.26–6.44)	1.82 (0.78–4.08)	0.88	
**APOA1 (rs670/rs5069)/APOC3 (rs5128)**						
**Sugar**						0.008
**GA+AA/CT+TT/CC (n=147)**	1	0.24 (0.07–0.87)	0.26 (0.06–1.02)	0.14 (0.04–0.52)	0.001	
**Others (n=659)**	0.24 (0.07–0.70)	0.32 (0.11–0.97)	0.22 (0.07–0.69)	0.32 (0.10–0.95)	0.55	
**Fish**						0.01
**GA+AA/CT+TT/CC (n=147)**	1	3.29 (1.09–9.92)	2.13 (0.61–7.33)	6.27 (1.86–21.2)	0.09	
**Others (n=659)**	2.24 (0.87–5.79)	3.32 (1.28–8.63)	3.00 (1.16–7.77)	1.78 (0.68–4.61)	0.98	
**Sweet beverages**						0.03
**GA+AA/CT+TT/CC (n=147)**	1	4.69 (1.46–15.08)	7.62 (2.19–26.53)	9.58 (2.70–33.94)	0.001	
**Others(n=659)**	2.98 (1.17–7.58)	3.93 (1.51–10.23)	4.07 (1.62–10.25)	4.41 (1.72–11.31)	0.009	

APOA1: rs670 (−75G>A), rs5069 (+83C>T), APOC3: rs5128 (3238C>G). DP: Dietary pattern, Q: Quartile of food group intakes.

1 ORs (95% CI) were calculated using conditional logistic regression model, adjusted for baseline BMI. Participants were joint classified (8 groups) according to quartiles of food group intakes and risk genotype carriers of rs670 (GA+AA) and rs5069 (CT+TT). The lowest quartile of food group intakes and risk genotype carriers were used as the reference group. When APOC3 rs5128 was included in the analysis, the risk genotype combination comprised rs670 (GA+AA), rs5069 (CT+TT) and rs5128 (CC).

## Discussion

The interaction between food group intakes and genetic variants of APOA1/APOC3 SNP (rs670, rs5069, rs5128) in relation with MetS risk was assessed in the present nested case-control study of Tehranian adults. Intake of sugar was associated with a lower risk of the MetS among the A allele carriers of rs670, T allele carriers of rs5069, the APOA1 combined GA+AA/CT+ TT/CC genotypes. Moreover, subjects with GG genotypes, in the highest quartiles of nuts consumption, in comparison with minor allele carriers with low nuts intake had the highest MetS risk. Sugar-sweet beverages (SSB) intakes interacted with combined APOA1/APOC3 genotypes on MetS risk. Intakes of SSB and fish groups were associated with higher MetS risk among individuals with combined GA+AA/CT+TT/CC genotypes.

The association between changes in the SSB consumption and the incidence of the MetS and its components was assessed in a Spanish Mediterranean cohort of university graduates ^30^. Participants who increased their consumption of SSB (upper *v*. lower quintile) after 6 years of follow-up, had a significantly higher risk of developing the MetS (adjusted OR 2.2, 95% CI: 1.4, 3.5; *P* for trend=0.003); in contrast to this study, the results of another Iranian study are in agreement with those of the current study ^31^. Khosravi-Boroujeni *et al* found no significant association between SSB intake and MetS, a lack of significant associations that persisted even after controlling for potential confounders. In addition to this study, Lutsey *et al* in a well-designed prospective study also showed that consumption of SSBs was not associated with incident MetS among middle-aged adults ^32^. Another study analyzing data from two well-kwon cohorts about SSB and genetic risk of obesity, assessed 32 polymorphisms that are known to be associated with BMI and observed that the higher the SSB intakes, the greater the BMI increase per increment of 10 risk allele ^33^.

Other studies have shown a direct correlation between sugar intake and risk of metabolic syndrome ^32,34,35^. One of the reasons that might explain why MetS risk was lower in the A allele carrier across the quartiles of sugar intake in the current study, is this fact that jam consumption has been considered in the definition of sugar group intake. Jams are made from different fruits rich in several antioxidants and polyphenols. Polyphenolic compounds are ubiquitous dietary components, mainly flavonoids and tannins. Specific polyphenols, effective in scavenging reactive oxygen and reactive nitrogen species, are able to modulate genes associated with metabolism, stress defense, drug metabolizing enzymes, and detoxification and transporter proteins. Their overall effect is protective in overcoming damaging effects of chronic diseases such as MetS ^36^. As mentioned above, another finding of the present study showed that consumption of nuts is related to MetS risk. Nuts as a whole are healthy foods because of their good fatty acid profile (low in saturated fats and high in mono- and polyunsaturated fats (MUFA and PUFA, respectively)) and low available carbohydrate content, as well as being good sources of vegetable protein, fiber, phytosterols, polyphenols, vitamins and minerals; nuts may therefore be a useful component of a dietary strategy aimed at improving the risk factors of the MetS, diabetes and CVD ^37^. Many studies have shown that nuts intake can improve glycemic control ^38–40^ and reduce serum LDL-C ^41,42^. It is possible that the beneficial effect of nuts on serum lipids and glucose may depend on the level of MUFA; however if the salty nuts are consumed, they can cause health risks such as increased blood pressure. Given that most of Iranian people consume nuts in the salty roasted form, it can be expected that the benefits of nuts consumption are covered by high intake of salt. On the other hand, nuts contain high energy density and high intakes of it may lead to overweight and obesity, which in turn, pave the way for the development of hypertension, hyperlipidemia and other risk factors of MetS.

Although interaction of APOA1 genotypes (rs670) and APOC3 (rs5128) with salty snacks and of APOA1 genotypes (rs670) with fast food were not significant, the increasing trend in consumption of these food groups was seen in subjects carrying the A and T alleles of ApoA1 and the CC homozygote of the APOC3 polymorphism. Fast foods have a high amount of saturated fat and trans-fatty acid, energy density, glycemic index, sodium, refined carbohydrate and therefore poor diet quality. Besides these negative points, the low dietary phytochemical index and total antioxidant capacity of mentioned food, fast foods have become one of the most important risk factors of MetS.

Some studies showed that carriers of the A allele were more responsive to changes in dietary fat than were GG subjects ^43,44^. Results of one study ^45^ examining the effect of fast food intake on the risk of MetS were consistent with those of the present study, and showed that after adjustment for all potential confounding variables, the risk of MetS, in the highest quartile of fast foods compared with the lowest, was 1.85 (95% CI:1.17–2.95). In the Qi *et al’s* study, interaction of fried food consumption with genetic risk in relation to BMI was assessed on 9623 women from the Nurses’ Health Study and 6379 men from the Health Professionals Follow-up Study. Interaction between fried food consumption and a genetic risk score was observed based on 32 BMI-associated variants in both the Nurses’ Health Study and Health Professionals Follow-up Study (p≤0.001 for interaction) ^46^.

Regarding the interaction of salty snack consumption and APOA1 genotype on MetS risk, it should be said that salty snack because of the high levels of salt can increase blood pressure over time. Although it is not clear how the mutant allele affects MetS risk, future studies are recommended to clarify the molecular mechanisms underlying these gene-nutrients interactions.

Participants with higher consumption of fish had a high risk of MetS, if they were the carriers of the T allele. Unlike many studies which show protective role of fish consumption on MetS risk, some studies demonstrated no association between fish consumption and risk of MetS ^47–49^. Fish preparation methods (*e.g*., frying) and certain contaminants in fish may attenuate the potential and beneficial effects of fish consumption. Since most Iranian people eat deep-fried fish, this may limit the protective effect of fish consumption on MetS, not seen in this population. In subjects with the major homozygote of APOA1 rs5069, higher quartiles of tea and coffee consumption were associated with a lower risk of MetS, compared with minor allele carriers. Coffee is among the most widely consumed beverages in the world ^50^, the consumption of coffee has been known to decrease the risk of type 2 diabetes ^51,52^. The favorite beverage of Iranians is tea, whereas coffee is not very common. Tea and coffee are important dietary sources of polyphenols (flavonols and catechins); because of high content of catechins (epicatechin, epicatechin gallate, epigallocatechin and epigallocat-echin gallate), also known as tea flavonoids, it may be assumed that the habitual consumption of tea seems to protect against the development of cardiovascular disease and inversely is associated with metabolic syndrome. Furthermore, several *in vitro* and *in vivo* studies have tried to elucidate the role of these beverages and a large amount of experimental studies clearly indicate beneficial effects of polyphenols on coronary artery disease. Moreover, the growing number of studies suggest that coffee consumption is associated with lower blood pressure ^53–55^, lower blood glucose ^51–53^ and lower triglycerides ^53^.

The main strength of the present study was its longitudinal design and the use of appropriate number of cases and matched controls from all demographic strata, and our results also emphasized the importance of taking into account the gene-diet interaction effect in association studies. Excluding subjects who created changes in dietary pattern because of having MetS was the strength of this study. Evaluation of dietary intake at just one time point was one of the limitations. However, assessing dietary intake by FFQ in about one year before developing MetS can cover this limitation. The second limitation was the lack of measurement of insulin sensitivity as a sensitive marker of MetS. For this reason, the present study did not detect any interaction between food groups and genotypes in relation to it. Another limitation of this study was considering the limited number of polymorphisms in the analysis of the interaction between dietary patterns and polymorphism in association with MetS that most polymorphisms in relation to the components of MetS do not cover. The small effect size of polymorphisms reduced the probability of observing a significant correlation. Future studies should investigate the genetic basis of individuals namely the cumulative effect of polymorphisms involved in the desired outcome and calculate their genetic risk score. Due to the limited sample size, there was no possibility of gender segregation in the analyses. Although lifestyle factors such as smoking, physical activity, education levels were considered in this study, inevitably some other confounding factors were not measured.

## Conclusion

In conclusion, this study provides evidence that some food groups modulate the effect of APOA1 and APOC3 SNPs in relation to MetS risk; being A and T allele carriers of APOA1 (rs670, rs5069) and C allele carriers of APOC3 (rs5128) further increased MetS risk in individuals with higher consumption of sweet beverages and fish groups. Also, A and T alleles of APOA1 appear to be protective for MetS risk in participants with higher scores of the sugar group intake.
